# Performance Analysis of Boosting-Based Machine Learning Models for Predicting the Compressive Strength of Biochar-Cementitious Composites

**DOI:** 10.3390/ma19020338

**Published:** 2026-01-14

**Authors:** Jinwoong Kim, Daehee Ryu, Heojeong Hwan, Heeyoung Lee

**Affiliations:** Department of Civil Engineering, Chosun University, 10 Chosundae 1-gil, Dong-Gu, Gwangju 61452, Republic of Korea; daw220@chosun.kr (J.K.); ryuhee99@chosun.ac.kr (D.R.); tzsfe@chosun.ac.kr (H.H.)

**Keywords:** biochar, cement substitute, machine learning, compressive strength prediction, boosting-based model, optimal model

## Abstract

**Highlights:**

**What are the main findings?**

- Biochar content and properties significantly affect compressive strength of cementitious composites.
- Optimal biochar dosages improve mechanical performance while supporting carbon reduction.
- Machine learning models accurately capture strength trends of biochar-modified composites.

**What are the implications of the main findings?**

- Biochar can be effectively used to design more sustainable cementitious materials.
- Data-driven models reduce experimental effort in strength prediction and mix optimization.
- Findings support low-carbon construction practices and performance-based material design.

**Abstract:**

Biochar, a carbon-rich material produced through the pyrolysis of wood residues and agricultural byproducts, has carbon storage capacity and potential as a low-carbon construction material. This study predicts the compressive strength of cementitious composites in which cement is partially replaced with biochar using machine learning models. A total of 716 data samples were analyzed, including 480 experimental measurements and 236 literature-derived values. Input variables included the water-to-cement ratio (W/C), biochar content, cement, sand, aggregate, silica fume, blast furnace slag, superplasticizer, and curing conditions. Predictive performance was evaluated using Multiple Linear Regression (MLR), Elastic Net Regression (ENR), Support Vector Regression (SVR), and Gradient Boosting Machine (GBM), with GBM showing the highest accuracy. Further optimization was conducted using XGBoost, Light Gradient-Boosting Machine (LightGBM), CatBoost, and NGBoost with GridSearchCV and Optuna. LightGBM achieved the best predictive performance (mean absolute error (MAE) = 3.3258, root mean squared error (RMSE) = 4.6673, mean absolute percentage error (MAPE) = 11.19%, and *R*^2^ = 0.8271). SHAP analysis identified the W/C and cement content as dominant predictors, with fresh water curing and blast furnace slag also exerting strong influence. These results support the potential of biochar as a partial cement replacement in low-carbon construction material.

## 1. Introduction

The global cement industry produces approximately four billion tons of cement annually, generating more than 2.6 billion tons of ${CO}_{2}$, accounting for approximately 7–8% of total anthropogenic ${CO}_{2}$ emissions [1,2,3]. In South Korea, the cement sector produces approximately 50 million tons of cement per year, and more than 12% of national ${CO}_{2}$ emissions originate from cement and concrete production [4]. The 2025 Carbon Neutrality Scenario designates biomass-based carbon storage technologies and low-carbon construction materials as key mitigation strategies [5]. The Third National Climate Change Adaptation Plan also identifies the expanded use of low-carbon materials and the development of circular construction resources as priority strategies [6]. Biochar is a carbon-rich material produced by pyrolyzing biomass such as wood residues and agricultural waste at 300–700 °C under oxygen-limited conditions. With carbon comprising 50–90% of the total mass and possessing a highly stable structure, biochar enables long-term carbon storage. Biochar production can reduce CO_2_ emissions by up to 80–90% compared with natural decomposition or open burning of biomass [7,8,9]. Due to these characteristics, the potential of biochar as a low-carbon construction material has been widely studied [10,11,12].

The applicability of biochar as a low-carbon additive in cementitious composites has been shown in numerous studies [13,14,15]. Zhao et al. [16] conducted a meta-analysis of the compressive strength of Portland cement composites incorporating biochar and that strength did not decrease when biochar content remained below 2.5 wt%. Qing et al. [17] investigated the compressive strength and fracture behavior of biochar concrete and observed strength enhancement at 1 wt% as well as improved crack resistance and load-bearing capacity at 3 wt%. Liu et al. [18] evaluated bamboo biochar as a partial replacement for cement in mortar and found that 1–3 wt% increased compressive strength and initial crack resistance compared with the control. Hylton et al. [19] replaced 10 wt% of cement with different biochars and indicated that compressive strength was maintained when the initial saturation percentage, W/C ratio, and soluble Si concentration fell within appropriate ranges. Akhtar et al. [20] assessed the mechanical performance of concrete incorporating poultry litter, rice husk, and pulp-and-paper sludge biochars and found that flexural and tensile strength were maintained at 1% cement replacement. Mo et al. [21] analyzed cement paste incorporating biochar and MgO expansive additive and a reduction in autogenous shrinkage and sustained internal relative humidity due to internal curing effects enabled by the porous biochar structure. Gupta and Kua [22] replaced 10 wt% of cement with biochar (wood-waste and food-waste) combined with silica fume and compressive strength increased by 18–20% compared with the control. These findings indicate that cementitious composites can maintain mechanical performance when appropriate types and proportions of biochar and admixtures are used, thereby confirming the potential of biochar as a low-carbon cement replacement.

Compressive strength is a fundamental indicator of structural performance in cementitious composites, and accurate evaluation of compressive strength is essential for safe structural design. However, cementitious composites are inherently heterogeneous systems composed of cement, sand, and supplementary components, which complicates reliable strength prediction. Conventional compressive strength testing requires specimen preparation and curing, resulting in significant time and cost demands. Moreover, repeated testing may produce inconsistent results, while nondestructive methods and empirical models often fail to capture the nonlinear behavior of cementitious composites [23,24,25]. Owing to these limitations, data-driven predictive models have attracted increasing attention. Machine learning has emerged as an effective approach because this approach can capture complex nonlinear interactions among multiple variables [26,27,28]. Recent studies have increasingly applied machine learning techniques to predict the compressive strength of cementitious materials [29,30,31]. For example, Huang et al. [32] used a hybrid machine learning model for cement-based materials containing metakaolin. The hybrid model achieved higher accuracy than single models. The water-to-binder ratio and metakaolin content were identified as key variables. Silva et al. [33] compared prediction models for Brazilian concrete and showed that Random Forest–based approaches provided stable predictions while effectively reflecting regional material characteristics. Wang et al. [34] predicted the compressive strength of conventional concrete using regression-based machine learning models, and eXtreme Gradient Boosting (XGBoost) achieved the highest performance with training $R^{2}=0.982$ and testing $R^{2}=0.966$. Feng et al. [35] utilized adaptive boosting to predict compressive strength and observed that AdaBoost outperformed individual regression models by effectively addressing nonlinear relationships. Nguyen et al. [36] predicted compressive and tensile strength using various machine learning methods, with gradient boosting and ensemble models yielding the lowest prediction errors and highest R^2^ values. Paixão et al. [37] evaluated four machine learning techniques for compressive strength prediction and indicated that Gaussian Process Regression and Artificial Neural Networks achieved the highest predictive accuracy. Elhishi et al. [38] applied interpretable artificial intelligence to assess variable importance, identifying cement content and the W/C ratio as dominant factors influencing compressive strength. Li et al. [39] trained a gradient boosting regression tree model using 1030 experimental data points and achieved $R^{2}=0.92$, outperforming $R^{2}=0.92$, single models. Le et al. [40] compared Deep Neural Network, K-Nearest Neighbors, and Support Vector Machine models for geopolymer concrete and found that the Deep Neural Network provided the highest accuracy, with the activator ratio and water-to-binder ratio identified as primary influencing variables. Collectively, these studies indicate that machine learning provides a robust framework for accurate compressive strength prediction, which is critical for ensuring structural performance and long-term durability in cementitious composites.

**Figure 1:**
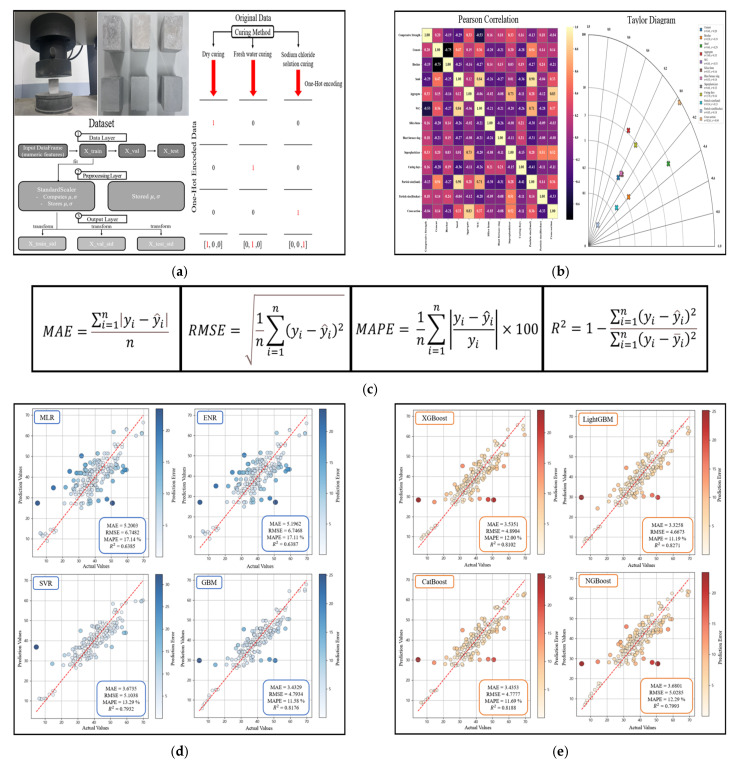
Research flowchart for predicting the compressive strength of biochar-cementitious composites. (**a**) Data preparation; (**b**) Correlation analysis; (**c**) Performance metrics; (**d**) Optimal machine learning model analysis; (**e**) Boosting-based machine learning model analysis.

This study applies machine learning techniques to predict the compressive strength of cementitious composites incorporating biochar as a partial replacement for cement. The overall research workflow is presented in Figure 1. Data preparation involved organizing the dataset into input and output variables specific to biochar-cementitious composites. During preprocessing, numerical variables were standardized, and categorical variables were converted using one-hot encoding [41,42,43]. Pearson correlation coefficients were used to examine relationships among input variables, and a Taylor diagram was used to visualize linear correlations and variance characteristics [44,45]. Model prediction performance was assessed using mean absolute error (MAE), root mean squared error (RMSE), mean absolute percentage error (MAPE), and $R^{2}$. Optimal algorithm selection was performed by comparing linear models (Multiple Linear Regression (MLR) and Elastic Net Regression (ENR)), a nonlinear kernel-based model (Support Vector Regression (SVR)), and a boosting-based model (Gradient Boosting Machine (GBM)). The optimal algorithm was subsequently extended to advanced boosting-based models, including XGBoost, Light Gradient-Boosting Machine (LightGBM), Categorical Boosting (CatBoost), and Natural Gradient Boosting (NGBoost). Hyperparameter optimization was conducted to further improve predictive performance. Based on comprehensive comparative analyses, the most effective model for predicting the compressive strength of biochar-cementitious composites was identified. Finally, SHAP analysis was performed to quantify the sensitivity and relative influence of input variables on model predictions. This paper is organized as follows. Section 2 describes the dataset composition, the definition of input and output variables, and the data preprocessing procedures. Section 3 presents the correlation analysis, machine learning methodologies, model training process, and performance evaluation. Section 4 discusses the comparative results and provides SHAP-based interpretation of variable contributions. Section 5 presents the main findings and conclusions.

## 2. Experimental Program

### 2.1. Experimental Process

**Figure 2:**
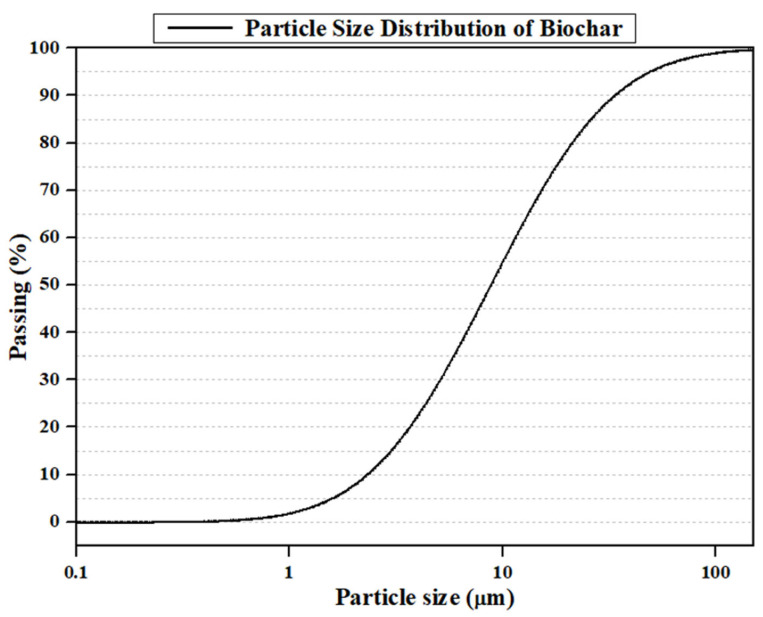
Particle size distribution of biochar.

**Figure 3:**
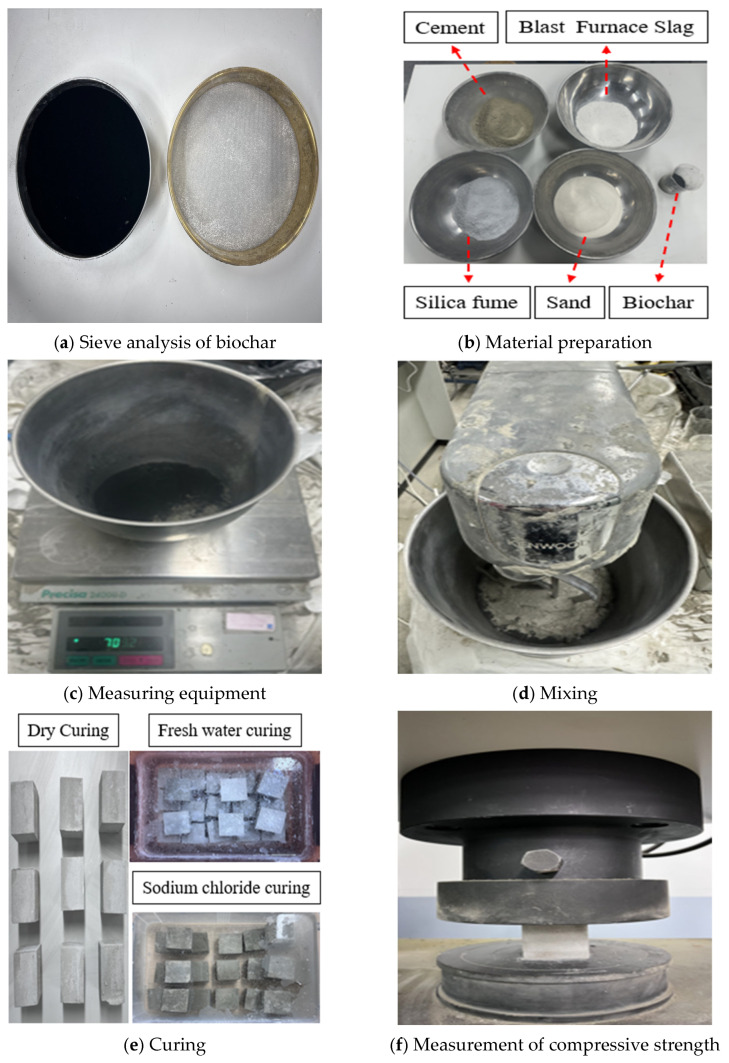
Experimental process.

Figure 2 presents the particle size distribution of the biochar used in this study [46,47]. Table 1 shows the elemental composition of the biochar, which represents the chemical characteristics of the material. The biochar was produced through three sequential stages, namely natural drying, oven drying at 80 °C, and pyrolysis at 500 °C. The experimental procedure for evaluating the compressive strength of biochar-cementitious composites is shown in Figure 3. To ensure a homogeneous particle size distribution, the biochar was sieved using a No. 50 sieve with a sieve opening size of 300 μm (Figure 3a). Figure 3b shows the materials used in producing the biochar-cementitious composites. Type I Portland cement was used, and the admixtures satisfied relevant ASTM standards ASTM C1240-20 and ASTM C989-19 [48,49]. Specimen fabrication began with weighing cement, biochar, sand, and admixtures according to the specified mix proportions (Figure 3c). The measured materials were placed in a mixing bowl (Kenwood, Havant, UK) and blended using a mechanical mixer (Figure 3d). The resulting biochar-cementitious composites were cast into molds measuring 50 mm × 50 mm × 50 mm [50]. As shown in Figure 3e, the specimens were cured under three conditions: dry curing, fresh water curing, and sodium chloride curing [51]. Finally, compressive strength was measured using a 1000 kN capacity universal testing machine (UTM, Daekyung Tech, Incheon, Republic of Korea) [52]. A loading rate of 1 mm/min was applied, and compressive strength was measured in MPa [53]. All compressive strength tests were conducted in accordance with ASTM C109 (Figure 3f) [54].

### 2.2. Datasets

**Figure 4:**
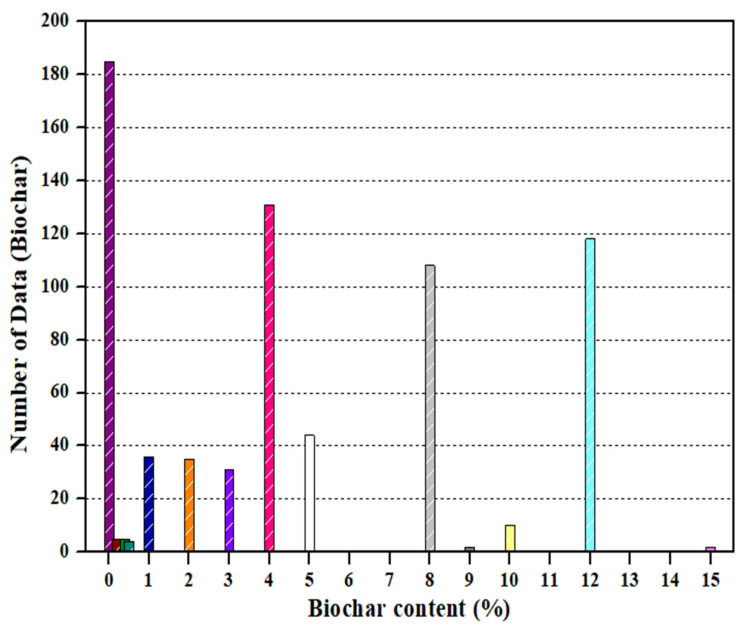
Distribution of biochar content in the dataset.

**Figure 5:**
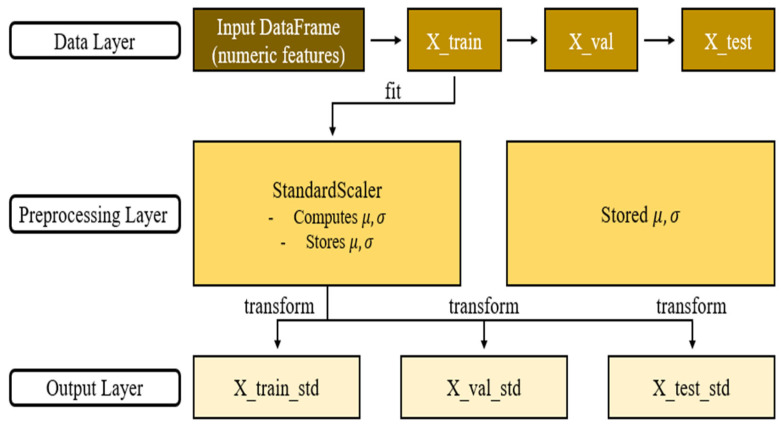
Standardization process of the dataset.

**Figure 6:**
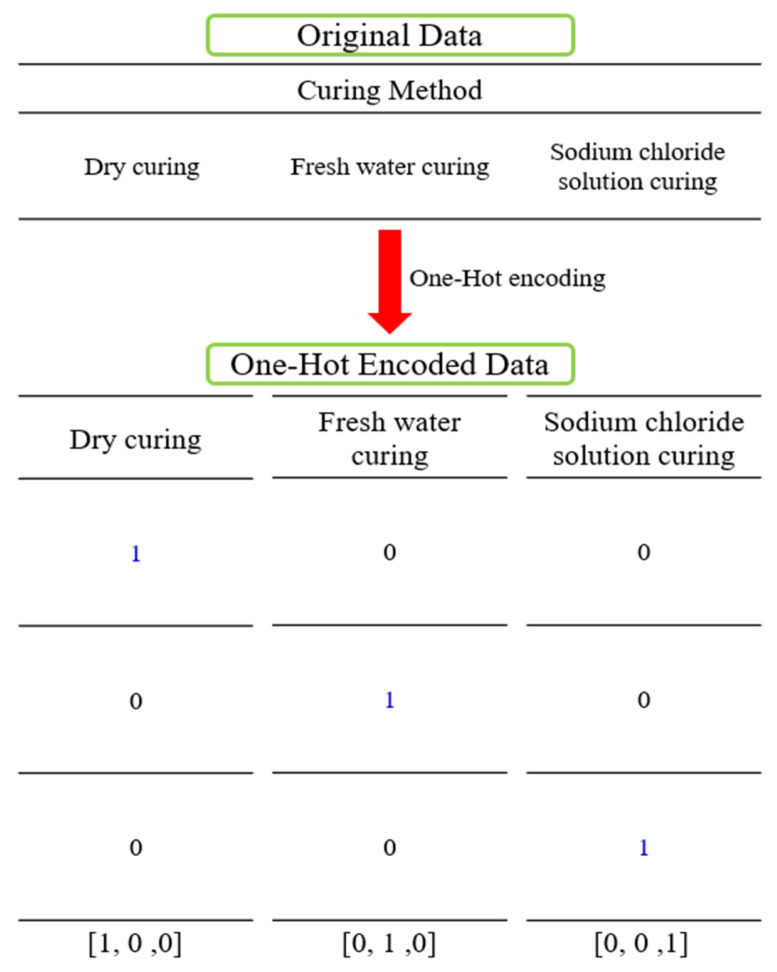
One-hot encoding process [26].

Compressive strength is a key indicator for ensuring the structural performance and stability of cementitious composites and was therefore selected as the output variable. A total of 716 data points were used in this study, comprising 480 experimental measurements and 236 values obtained from the literature (Supplementary Material File). Input variables included cementitious composite components such as cement, biochar, sand, aggregate, and the W/C ratio, as well as admixtures including silica fume, blast furnace slag, and superplasticizer. Curing days were incorporated to reflect long-term strength development. In addition, curing conditions, including dry curing, fresh water curing, and sodium chloride curing, were included to reflect environmental influences. Particle size (for sand and biochar) and cross-sectional area were included to account for material characteristics (Table 2). The biochar replacement range investigated in this study (0–15%) was selected based on values documented in the literature. Rather than defining an optimal biochar dosage under specific curing conditions, this study focused on analyzing overall trends and predictive patterns within this literature-based range (Figure 4). The references for the biochar-cementitious composite compressive strength dataset are listed in Table 3. During preprocessing, standardization was applied to reduce scale differences among variables and enhance model training stability (Figure 5). Categorical variables were transformed using one-hot encoding to produce binary vectors (Figure 6). For example, the three curing conditions, namely dry curing, fresh water curing, and sodium chloride curing, were encoded using True (1) and False (0) values. This procedure improves the ability of the model to learn from both continuous and categorical variables [55,56,57]. The dataset was divided into training and test sets using a 70/30 split.

## 3. Research Method

### 3.1. Correlation Analysis

Pearson correlation is used to evaluate the linear relationship between two continuous variables, and the correlation coefficient ranges from −1 to +1. A value close to +1 indicates a strong positive linear correlation, while a value close to −1 indicates a strong negative linear correlation. As shown in Equation (1), the Pearson correlation coefficient is calculated by dividing the covariance of the two variables by the product of the standard deviations of the two variables. The Taylor diagram provides a simultaneous visualization of the correlation and variance structure between predicted and observed values. This visualization complements Pearson correlation analysis by revealing correlation strength together with distributional characteristics of variables [66,67,68].

(1) $$r=\frac{\sum_{i=1}^{n}\left(x_{i}-\bar{x}\right)\left(y_{i}-\bar{y}\right)}{\sqrt{\sum_{i=1}^{n}{\left(x_{i}-\bar{x}\right)}^{2}}\sqrt{\sum_{i=1}^{n}{\left(y_{i}-\bar{y}\right)}^{2}}}$$

In this equation, r represents the Pearson correlation coefficient between variables x and y, n is the number of data points, $x_{i}$ and $y_{i}$ denote the individual data values, and $\bar{x}$ and $\bar{y}$ are the mean values of each variable.

**Figure 7:**
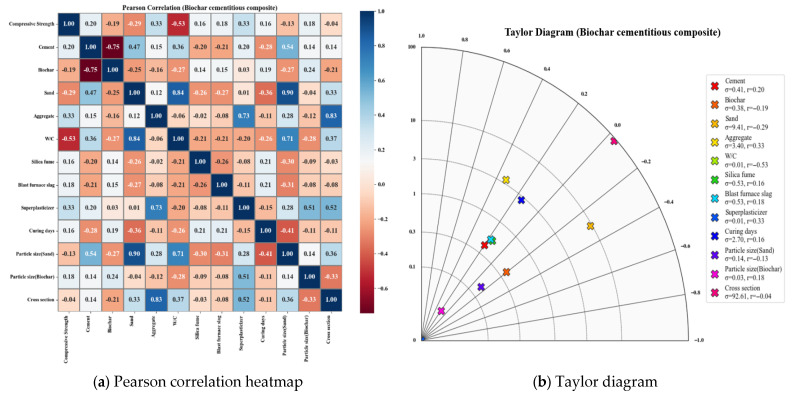
Correlation analysis results.

The Pearson correlation analysis indicated that biochar (0.38) and cement (0.41) exhibited positive correlations with compressive strength (Figure 7a). This tendency is attributed to the filler effect of fine biochar particles and the promotion of hydration reactions, both of which contribute to strength enhancement. Cement content also showed a positive relationship with compressive strength due to the formation of calcium-silicate-hydrate (C–S–H), which is the primary contributor to strength increase. Sand (0.47) and aggregate (0.41) also exhibited positive correlations, indicating that higher aggregate proportions contribute to strength improvement. By contrast, the W/C (−0.62) exhibited the strongest negative correlation, reflecting the well-established effect of increased water content in reducing compressive strength in cementitious composites. Superplasticizer (0.18) showed a weak positive correlation, whereas silica fume (−0.16) and blast furnace slag (0.05) exhibited low correlation values. Curing days (0.27) showed a positive correlation, indicating that longer curing durations contribute to strength improvement.

The Taylor diagram further supported these observations. Cement and sand showed relatively high correlation coefficients and stable variance, consistent with the Pearson correlation results (Figure 7b). The W/C revealed negative correlation and high variance, reaffirming the strong influence of the W/C on strength reduction. Biochar presented a moderate correlation coefficient and stable variance, indicating a consistent contribution to compressive strength. Curing days showed a distribution consistent with the Pearson correlation results, confirming that extended curing enhances strength development.

### 3.2. Machine Learning Approaches

**Figure 8:**
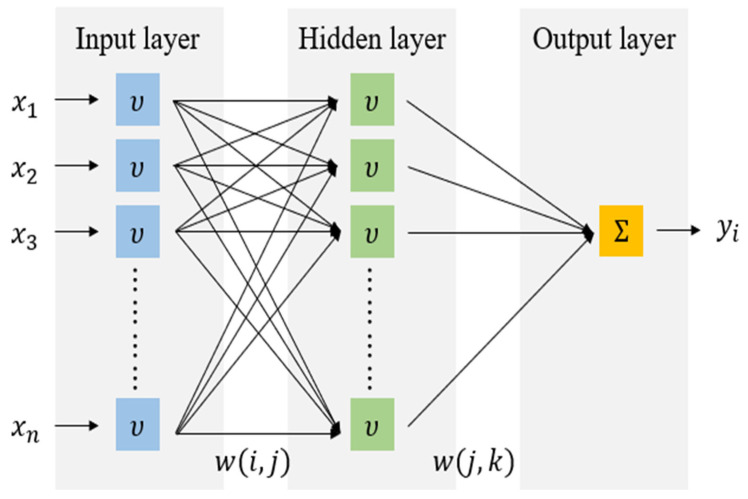
MLR architecture.

**Figure 9:**
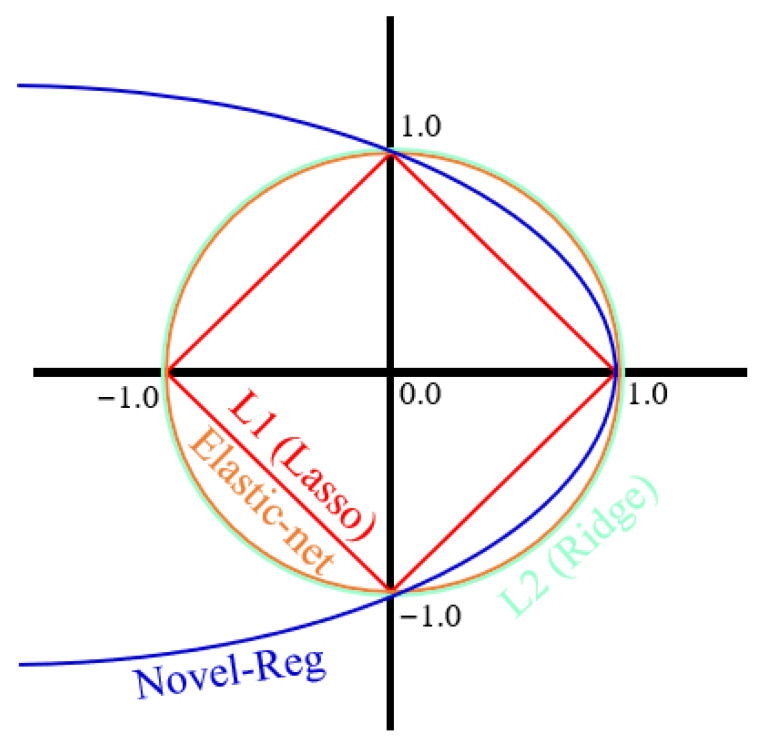
ENR architecture.

**Figure 10:**
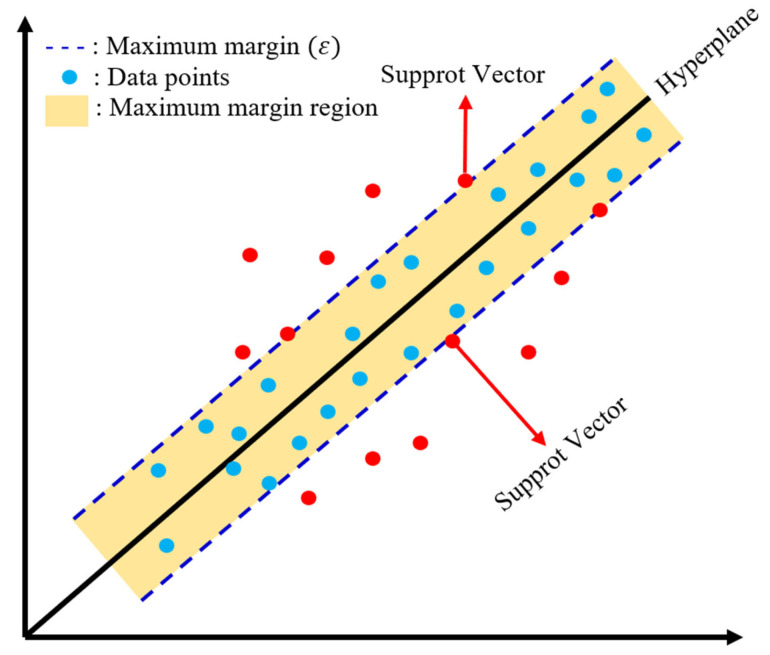
SVR architecture.

**Figure 11:**
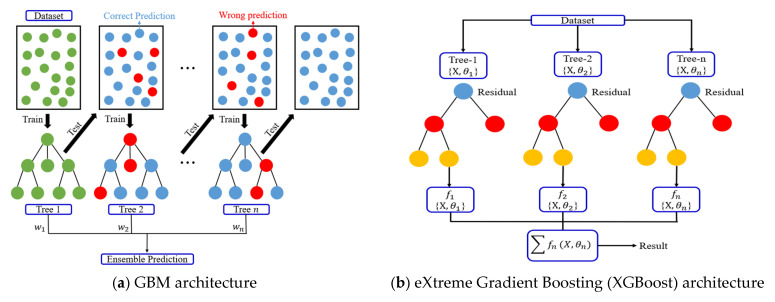

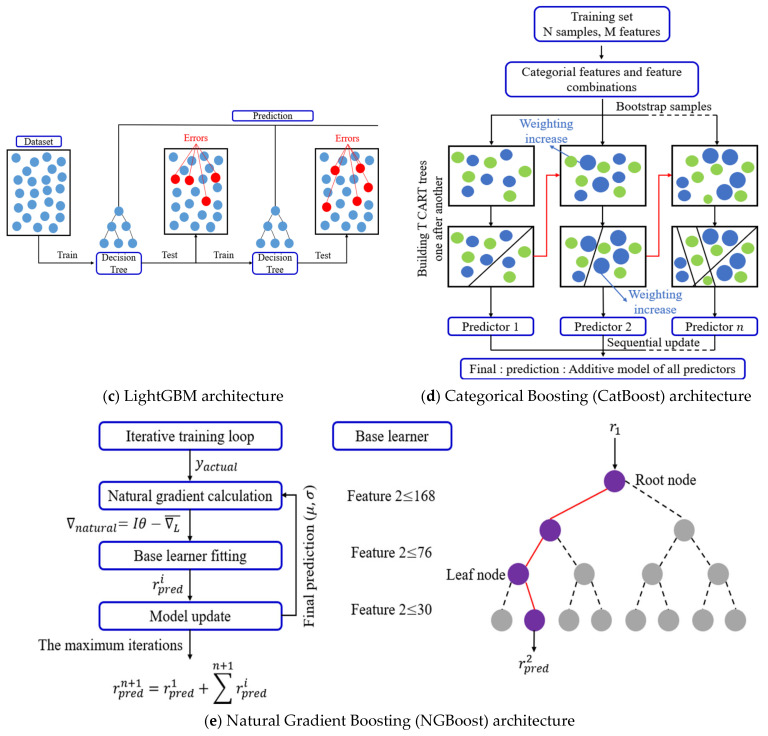
Architecture of boosting-based machine learning models.

This study used linear, nonlinear, and boosting-based models to predict the compressive strength of biochar-cementitious composites. Optimal algorithm selection was performed by comparing representative models from each category, and the main characteristics of these models are described below. MLR predicts compressive strength by modeling linear relationships between input variables and output values. MLR offers a simple model structure and straightforward interpretability, allowing direct identification of the linear influence of each variable [69]. However, MLR is sensitive to multicollinearity, and prediction performance can degrade when strong correlations exist among input variables (Figure 8). ENR is a regression method that simultaneously applies L1 and L2 regularization [69]. The L1 component facilitates variable selection, whereas the L2 component stabilizes regression coefficients. By integrating these properties, ENR provides robust predictions in regression problems affected by multicollinearity and yields more stable coefficients under correlated input conditions. Figure 9 shows the ENR architecture. SVR models nonlinear relationships between input and output variables by mapping data into a high-dimensional feature space [70]. Through kernel functions, SVR captures complex nonlinear patterns that linear models cannot represent. SVR also follows the principle of structural risk minimization, which reduces overfitting and supports stable prediction performance (Figure 10). GBM is a boosting-based regression method that sequentially combines weak learners to iteratively reduce prediction errors. GBM enhances predictive capability by training each subsequent learner on the residuals of the previous model [71]. This stage-wise learning framework improves the ability to capture complex input relationships while controlling overfitting and maintaining stable predictive accuracy (Figure 11a).

Based on the identified optimal algorithm, additional performance evaluations were conducted using advanced boosting-based models. XGBoost is an enhanced boosting method that incorporates second-order derivative information to minimize the loss function. XGBoost iteratively learns residual errors to achieve high predictive performance and includes regularization components to mitigate overfitting (Figure 11b). XGBoost also supports distributed parallel processing and optimized tree structures, enabling high computational efficiency for large datasets [72]. LightGBM is a gradient boosting-based model that adopts a leaf-wise growth strategy [72]. LightGBM utilizes histogram-based learning to reduce computational cost while efficiently constructing deep tree structures, resulting in fast training speeds even for large-scale datasets. The architecture of LightGBM is shown in Figure 11c. CatBoost is designed to effectively handle categorical variables within a boosting framework. CatBoost applies target-based encoding and an ordering mechanism to minimize information loss from categorical variables and reduce overfitting (Figure 11d). A symmetric tree structure enables stable learning and provides effective modeling of complex nonlinear relationships [73]. NGBoost is a boosting model that estimates predictive distributions rather than point estimates [74]. By applying natural gradients, NGBoost stabilizes parameter updates and facilitates predictive uncertainty estimation. NGBoost can also incorporate various probability distributions, making NGBoost suitable for capturing complex data characteristics. The architecture of NGBoost is shown in Figure 11e. The optimal detailed model was selected by comparing the predictive performance of linear, nonlinear, and boosting-based approaches for estimating the compressive strength of biochar-cementitious composites. The mathematical formulations of the models used in this study are presented in Table 4. All machine learning analyses were conducted using Python (v3.9). Linear and nonlinear models, including MLR, ENR, and SVR, were implemented using the scikit-learn library, whereas boosting-based models were implemented using XGBoost (v1.7.6), LightGBM (v4.1.0), CatBoost (v1.2), and NGBoost (v0.5.3). These widely used open-source libraries provide validated implementations and ensure the reliability and reproducibility of the computational results.

### 3.3. Model Evaluation Metrics

The predictive performance of the biochar-cementitious composite compressive strength models was evaluated using MAE, RMSE, MAPE, and the coefficient of determination ($R^{2}$). MAE and RMSE approach 0 as prediction error decreases, whereas MAPE expresses relative error as a percentage, with lower values indicating higher predictive accuracy [75,76,77]. $R^{2}$ values closer to 1 indicate greater explanatory power. The formulas for these metrics are provided below [78].

MAE is calculated as the mean of the absolute differences between actual and predicted values (Equation (2)).

(2) $$MAE=\frac{1}{n}\sum_{i=1}^{n}\left|y_{i}-\hat{y_{i}}\right|$$

Here, n denotes the number of data points, $y_{i}$ is the actual value, and ${\hat{y}}_{i}$ is the predicted value.

RMSE is the square root of the mean of squared errors and indicates the magnitude of prediction error (Equation (3)). As the square root of the mean squared error (MSE), RMSE reflects the average magnitude of deviations between actual and predicted values.

(3) $$RMSE=\sqrt{\frac{1}{n}\sum_{i=1}^{n}{\left(y_{i}-\hat{y_{i}}\right)}^{2}}$$

MAPE is calculated by dividing the absolute error by the actual value for each observation and averaging the resulting percentages, thereby expressing the prediction error as a percentage (Equation (4)).

(4) $$MAPE=\frac{1}{n}\sum_{i=1}^{n}\left|\frac{y_{i}-{\hat{y}}_{i}}{y_{i}}\right|\times 100$$

$R^{2}$ represents the proportion of variance in the dependent variable explained by the independent variables (Equation (5)).

(5) $$R^{2}=1-\frac{\sum_{i=1}^{n}{\left(y_{i}-\hat{y_{i}}\right)}^{2}}{\sum_{i=1}^{n}{\left(y_{i}-\bar{y}\right)}^{2}}$$

In this formula, n is the number of data points, $y_{i}$ is the actual value, ${\hat{y}}_{i}$ is the predicted value, and $\bar{y}$ denotes the mean of the actual values.

## 4. Result and Discussion

### 4.1. Comparison of Machine Learning Models and Optimal Model Selection

**Figure 12:**
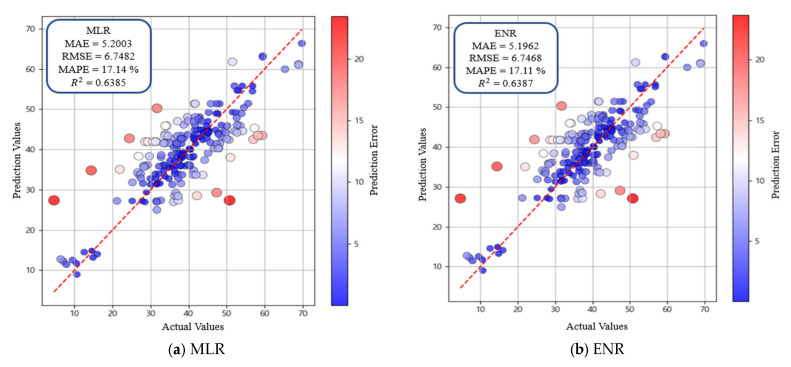

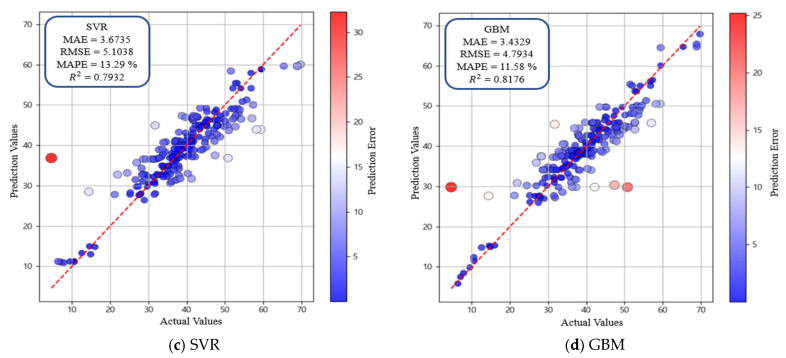
Performance evaluation of optimal machine learning models. The red dashed line represents the y = x reference line indicating perfect agreement between predicted and actual values, and the color scale denotes the prediction error.

Table 5 presents the predictive performance of the MLR, ENR, SVR, and GBM models evaluated on the independent test set. MLR achieved the lowest predictive accuracy, with MAE = 5.2003, RMSE = 6.7482, MAPE = 17.14%, and $R^{2}$ = 0.6385 (Figure 12a). This outcome indicates that linear regression models do not adequately capture the nonlinear characteristics inherent in the biochar-cementitious composite dataset. Although ENR incorporates both L1 and L2 regularization, ENR performance was similar to MLR performance (Figure 12b). By contrast, SVR showed improved performance by modeling nonlinear relationships through kernel-based learning (MAE = 3.6735, RMSE = 5.1038, MAPE = 13.29%, and $R^{2}$ = 0.7932). This finding indicates that SVR was the second-best-performing model during the optimal algorithm selection stage (Figure 12c). GBM exhibited the highest predictive accuracy, with MAE = 3.4329, RMSE = 4.7934, MAPE = 11.58%, and $R^{2}$ = 0.8176 (Figure 12d). This performance improvement is attributed to the boosting structure, which iteratively learns residuals and captures nonlinear relationships and interactions among variables. The tree-based splitting process further enhances predictive accuracy by reflecting the combined effects of multiple variables. Based on these results, the boosting-based GBM outperformed the other regression models. Consequently, additional boosting-based models were evaluated in the subsequent analysis to identify the optimal model for predicting the compressive strength of biochar-cementitious composites.

### 4.2. Performance Analysis of Boosting Models

**Figure 13:**
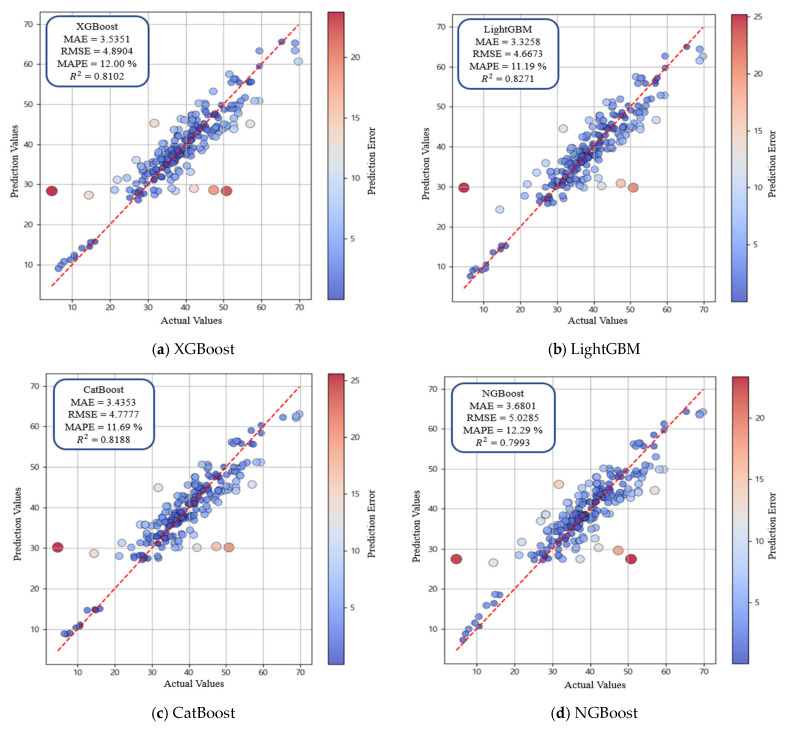
Performance comparison of boosting-based machine learning models. The red dashed line represents the y = x reference line indicating perfect agreement between predicted and actual values, and the color scale denotes the prediction error.

**Figure 14:**
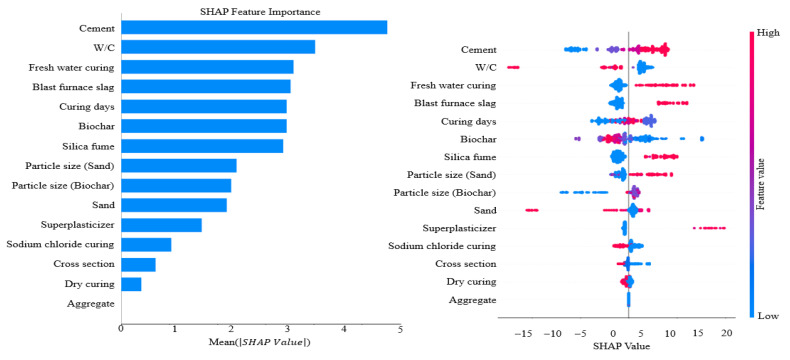
SHAP analysis results.

Boosting models were optimized using GridSearchCV (v1.2.2) and Optuna (v3.5.0) to identify optimal hyperparameter configurations [79,80]. GridSearchCV was performed using a 5-fold cross-validation approach, and the presented model performance corresponds to the optimized parameter set validated through repeated fitting. Each model tuned key parameters, including colsample_bytree, subsample, and max_depth, through cross-validation to improve generalization performance. The final optimized hyperparameters are presented in Table 6, and model performance after tuning is presented in Table 7. XGBoost learned complex patterns by iteratively fitting residuals, but predictive accuracy was lower than LightGBM predictive accuracy (MAE = 3.5351, RMSE = 4.8904, MAPE = 12.00%, $R^{2}=$ = 0.8102) (Figure 13a). LightGBM, which uses a leaf-wise splitting strategy, exhibited the best prediction performance among all boosting models (Figure 13b). LightGBM achieved MAE = 3.3258, RMSE = 4.6673, MAPE = 11.19%, and $R^{2}$ = 0.8271. CatBoost exhibited stable predictive capability owing to the ordering mechanism and robust handling of categorical variables, yielding performance comparable to XGBoost performance (Figure 13c). NGBoost ($R^{2}=$ 0.7993) incorporated a probabilistic boosting model that enabled estimation of prediction uncertainty and produced accuracy similar to that of SVR ($R^{2}=$ 0.7932), which was evaluated during the initial algorithm selection process (Figure 13d). Overall, these comparisons indicate that the leaf-wise structure of LightGBM is particularly effective for learning nonlinear patterns in the dataset, making LightGBM the optimal boosting model. SHAP analysis was then conducted to interpret variable contributions and analyze influence within the prediction process (Figure 14). Cement and the W/C exhibited the highest SHAP values, confirming dominant roles in determining compressive strength. Fresh water curing and blast furnace slag also showed high SHAP values, indicating substantial influence on model outputs. Curing days and biochar formed a second group of influential variables, indicating the importance of curing days and biochar in strength prediction. Silica fume and the two particle size variables (biochar and sand) showed moderate SHAP values with similar distributions. Superplasticizer and sodium chloride curing showed low SHAP values, whereas cross-sectional area, dry curing, and aggregate exhibited the lowest contributions. The SHAP distribution plot further revealed wide SHAP ranges for cement and W/C, while fresh water curing and blast furnace slag exhibited consistent patterns indicative of substantial model contribution. By contrast, cross-sectional area and aggregate showed narrow SHAP ranges, confirming limited influence on predictions. These findings indicate that SHAP analysis provides an effective framework for visualizing and interpreting both the magnitude and direction of variable influence and for clarifying the information structure used by the model during prediction. The strong influence of biochar content and particle size is primarily attributed to the high surface area and porous structure of biochar. These characteristics influence the interfacial transition zone by improving particle packing and promoting internal curing through moisture retention, which contributes to strength development in cementitious composites. Consequently, the SHAP-based feature importance reflects not only statistical significance but also the underlying physical mechanisms governing material behavior.

## 5. Conclusions

A total of 716 datasets, including 480 experimental measurements and 236 literature-derived values, were utilized to predict the compressive strength of biochar-cementitious composites. The dataset was processed using standardization and one-hot encoding to construct input and output variables. Correlation analysis was conducted using Pearson correlation and a Taylor diagram. Optimal algorithm selection consisted of evaluating linear models (MLR, ENR), a nonlinear model (SVR), and a boosting-based model (GBM). Based on the optimal algorithm, advanced boosting-based models, including XGBoost, LightGBM, CatBoost, and NGBoost, were further analyzed. Finally, SHAP analysis was applied to interpret the magnitude and direction of variable influence and to examine the information structure used during prediction.

- Pearson correlation analysis showed that biochar (0.38) and cement (0.41) exhibited positive correlations with compressive strength, and similar trends were observed for sand (0.47) and aggregate (0.41). By contrast, the W/C (−0.62) revealed a strong negative correlation, indicating the dominant role of the W/C in strength decrease. Superplasticizer (0.18) showed a weak positive correlation, and silica fume (−0.16) and blast furnace slag (0.05) exhibited low correlation values. These tendencies were consistent with the Taylor diagram results, where biochar and curing days (0.27) presented moderate correlations and stable variance, indicating a consistent influence on compressive strength.
- The comparison of linear, nonlinear, and boosting-based models revealed that MLR had the lowest predictive performance (with $R^{2}$ = 0.6385), and ENR exhibited comparable accuracy. SVR partially reflected nonlinear relationships and achieved improved performance ($R^{2}$ = 0.7932). GBM provided the highest accuracy ($R^{2}$ = 0.8176) by effectively modeling nonlinearities and variable interactions through a residual-based boosting structure. These results indicate that GBM is more stable and better suited for predicting the compressive strength of biochar-cementitious composites than the other evaluated models.
- Additional comparisons were conducted among boosting-based models, including XGBoost, LightGBM, CatBoost, and NGBoost. Hyperparameter tuning using GridSearchCV and Optuna improved predictive performance. LightGBM achieved the highest accuracy among all models and was identified as the optimal detailed model for compressive strength prediction (MAE = 3.3258, RMSE = 4.6673, MAPE = 11.19%, and $R^{2}$ = 0.8271). XGBoost and CatBoost showed lower accuracy than LightGBM, while maintaining stable prediction performance. NGBoost ($R^{2}$ = 0.7993) estimated predictive uncertainty through its probabilistic boosting framework and exhibited accuracy comparable to that of SVR ($R^{2}$ = 0.7932). Overall, LightGBM most effectively learned nonlinear data patterns and was selected as the optimal boosting-based model for predicting the compressive strength of biochar-cementitious composites.
- SHAP analysis indicated that cement and the W/C had the highest SHAP values, showing the dominant influence of cement and the W/C on compressive strength prediction. Fresh water curing and blast furnace slag also exhibited high SHAP values, achieved significant contributions to model output. Curing days and biochar formed a second group of influential variables. Silica fume and both particle size variables (biochar and sand) showed moderate SHAP values, whereas superplasticizer and sodium chloride curing exhibited low influence. Cross-sectional area, dry curing and aggregate had the smallest SHAP values. The SHAP distribution plots confirmed the strong impact of cement and the W/C, and the consistent distributions of fresh water curing and blast furnace slag further indicated the importance of fresh water curing and blast furnace slag in the prediction process. These results indicate that biochar has potential as a low-carbon construction material when used as a partial cement replacement.
- Compared with conventional compressive strength tests that require specimen preparation and curing time, the machine learning approach enables efficient evaluation of compressive strength after model training. This approach reduces the time and effort associated with experimental testing and facilitates performance assessment of biochar-cementitious composites.
- This study predicted the compressive strength of biochar-cementitious composites using machine learning to evaluate the potential of biochar as a low-carbon construction material. Future research will focus on predicting long-term strength, flexural strength, freeze–thaw durability, fire resistance, and other performance characteristics of biochar-cementitious composites under diverse service conditions.

## Figures and Tables

**Table 1:** Elemental composition of the biochar.

Component	Weight Ratio (%)
Carbon (C)	82.12
Oxygen (O)	16.02
Calcium (Ca)	0.97
Potassium (K)	0.89

**Table 2:** Input variables with units and ranges for biochar cementitious composites.

Variables	Unit	Range of Data
Cement	%	88–100
Biochar		0–15
Sand		0–465
Aggregate		0–254
Silica fume		0–20
Blast furnace slag		0–15
Superplasticizer		0.0–2.5
Water-to-cement (W/C)	-	0.28–0.45
Curing day	day	1–91
Particle size	μm	0.15–5
Cross section	${mm}^{2}$	1600–7854
Curing condition	-	Dry curing
		Fresh water curing
		Sodium chloride curing

**Table 3:** Reference compressive strength of biochar cementitious composites.

Index	Data Count	Reference
482–497	16	[58]
498–512	15	[59]
513–522	10	[60]
523–534	12	[61]
535–542	8	[62]
543–576	34	[63]
577–611	35	[18]
612–621	10	[64]
622–717	96	[65]

**Table 4:** Mathematical formulations of the machine learning models used in this study.

Model	Mathematical Formulation
MLR	y=Xβ+ε
ENR	$min\beta \;{\left\|y-X\beta \right\|}_{2}^{2}+\lambda \left(\alpha {\left\|\beta \right\|}_{1}+\left(1-\alpha \right){\left\|\beta \right\|}_{2}^{2}\right)$
SVR	${min}_{w,b,\xi ,\xi^{*}}\;\frac{1}{2}{\left\|w\right\|}^{2}+C\sum_{i=1}^{n}\left(\xi_{i}+\xi_{i}^{*}\right)\;subject\;to\;y_{i}-\left(w^{⊺}\emptyset \left(x_{i}\right)+b\right)≦\;\varepsilon +\xi_{i,}\;\left(w^{⊺}\emptyset \left(x_{i}\right)+b\right)-y_{i}\;≦\;\varepsilon +\xi_{i}^{*}$
GBM	$F_{m}\left(x\right)=F_{m-1}\left(x\right)+r_{m}h_{m}(x)$
XGBoost	$L=\sum_{i}l\left(y_{i,}{\hat{y}}_{i}\right)+\sum_{k}\Omega \left(f_{k}\right),\;where\;\Omega \left(f\right)=\gamma T+\frac{1}{2}\lambda {\left\|w\right\|}^{2}\;$
LightGBM	$L=\sum_{i}l\left(y_{i,}{\hat{y}}_{i}\right)+\sum_{k}\Omega \left(f_{k}\right)\;(leaf\;wise\;tree\;growth\;with\;regularization)$
CatBoost	$L=\sum_{i}l(y_{i},{\hat{y}}_{i}^{\left(t\right)})\;with\;ordered\;boosting\;to\;reduce\;prediction\;shift$
NGBoost	$L=\sum_{i}S\left(\theta \left(x_{i}\right),y_{i}\right),\;where\;S\;is\;a\;proper\;scoring\;rule\;and\;$ θ are distribution parameters

**Table 5:** Performance evaluation of optimal machine learning models.

Machine Learning Model	MAE	RMSE	MAPE (%)	$R^{2}$
MLR	5.2003	6.7482	17.14	0.6385
ENR	5.1962	6.7468	17.11	0.6387
SVR	3.6735	5.1038	13.29	0.7932
GBM	3.4329	4.7934	11.58	0.8176

**Table 6:** Hyperparameter settings of boosting-based machine learning models.

XGBoost	colsample_bytree	0.75
	learning_rate	0.03
	max_depth	4
	min_child_weight	5
	n_estimators	200
	reg_alpha	0.0
	reg_lambda	1.0
	subsample	0.6
LightGBM	colsample_bytree	0.6
	learning_rate	0.05
	max_depth	5
	n_estimators	200
	num_leaves	15
	reg_lambda	0.0
	subsample	0.6
CatBoost	depth	5
	l2_leaf_reg	5
	learning_rate	0.03
	min_data_in_leaf	3
	n_estimators	350
	subsample	0.6
NGBoost	n_estimators	300
	learning_rate	0.02916
	minibatch_frac	0.3223
	col_sample	0.5986

**Table 7:** Performance comparison of boosting-based machine learning models.

Machine Learning Model	MAE	RMSE	MAPE (%)	$R^{2}$
XGBoost	3.5351	4.8904	12.00	0.8102
LightGBM	3.3258	4.6673	11.19	0.8271
CatBoost	3.4353	4.7777	11.69	0.8188
NGBoost	3.6801	5.0285	12.29	0.7993

## Data Availability

The original contributions presented in the study are included in the article/Supplementary Material. Further inquiries can be directed to the corresponding author.

## Supplementary Materials

The following supporting information can be downloaded at: https://www.mdpi.com/article/10.3390/ma19020338/s1, Table S1: Summary of the dataset used in this study (716 data points).

## Abbreviations

The following abbreviations are used in this manuscript:

W/C	water-to-cement ratio
SF	Silica fume
BFS	Blast furnace slag
MLR	Multiple linear regression
ENR	Elastic net regression
SVR	Support vector regression
GBM	Gradient boosting machine
XGBoost	eXtreme Gradient Boosting
LightGBM	Light Gradient Boosting Machine
Catboost	Categorical boosting
NGBoost	Natural gradient boosting
MAE	Mean absolute error
RMSE	Root mean squared error
MAPE	Mean absolute percentage error
